# Reply to Rossettini et al. “Do Not Mix Apples with Oranges” to Avoid Misinterpretation of Placebo Effects in Manual Therapy: The Risk Is Resulting in a Fruit Basket. Comment on “Molina-Álvarez et al. Manual Therapy Effect in Placebo-Controlled Trials: A Systematic Review and Meta-Analysis. *Int. J. Environ. Res. Public Health* 2022, *19*, 14021”

**DOI:** 10.3390/ijerph20156445

**Published:** 2023-07-26

**Authors:** Miguel Molina-Álvarez, Alberto Arribas-Romano, Carmen Rodríguez-Rivera, Miguel M. García, Josué Fernández-Carnero, Susan Armijo-Olivo, Carlos Goicoechea Garcia

**Affiliations:** 1 Escuela Internacional de Doctorado, Faculty of Health Sciences, Universidad Rey Juan Carlos, 28922 Alcorcón, Spain; miguel.molina@urjc.es (M.M.-Á.); alberto.arribas@urjc.es (A.A.-R.); 2 Area of Pharmacology, Nutrition and Bromatology, Department of Basic Health Sciences, Rey Juan Carlos University, Unidad Asociada I+D+i Instituto de Química Médica (IQM) CSIC-URJC, 28922 Alcorcón, Spain; carmen.rodriguez@urjc.es (C.R.-R.); miguelangel.garcia@urjc.es (M.M.G.); carlos.goicoechea@urjc.es (C.G.G.); 3 Department of Physical Therapy, Occupational Therapy, Rehabilitation and Physical Medicine, Rey Juan Carlos University, 28922 Alcorcón, Spain; 4 High Performance Experimental Pharmacology Research Group, Rey Juan Carlos University (PHARMAKOM), 28922 Alcorcón, Spain; 5 Grupo Multidisciplinar de Investigación y Tratamiento del Dolor, Grupo de Excelencia Investigadora URJC-Banco de Santander, 28922 Alcorcón, Spain; 6 La Paz Hospital Institute for Health Research, IdiPAZ, 28029 Madrid, Spain; 7 Faculty of Business and Social Sciences, University of Applied Sciences, 30A, 49076 Osnabruck, Germany; susanarmijo@gmail.com; 8 Faculties of Rehabilitation Medicine and Medicine and Dentistry, 3-48 Corbett Hall, Edmonton, AB T6G 2G4, Canada

We have thoroughly reviewed and carefully analyzed the points raised in the comment titled: “Do not mix apples with oranges” to avoid misinterpretation of placebo effects in manual therapy: the risk is resulting in a fruit basket [1]. We sincerely appreciate the authors’ interest in the topic and their valuable contribution to the ongoing discourse on enhancing placebo groups in manual therapy trials. While we acknowledge that many of the limitations highlighted by the authors have already been discussed in the manuscript [2], we would like to address specific points for further discussion.

Physiotherapy and pain treatment encounter significant heterogeneity due to challenges in diagnosing specific pain conditions and the lack of treatment standardization. However, initial meta-analyses elucidating effective treatments across different conditions, despite variations in treatment dosage and patient characteristics, have greatly contributed to knowledge development. Therefore, our meta-analysis serves as a valuable resource for researchers, underscoring the importance of carefully selecting an appropriate control group for their studies. Moreover, it provides clinicians with insights into the pivotal role that placebo groups play in drawing meaningful conclusions from clinical trials of manual therapy.

Regarding the inclusion of various medical conditions, we were aware of this potential limitation, and we duly noted that caution must be exercised when interpreting the results. However, we opted not to perform subgroup analysis due to limited number of studies available for analysis. Furthermore, our study specifically focused on short-term responses in self-reported pain. It has been suggested that the mechanical stimulus initiates a cascade of neurophysiological effects that contribute to the pain-inhibitory response of manual therapy [3,4]. Therefore, while we acknowledge that the specific condition might have some significance, it is not necessarily the sole determining factor for the observed response.

We recognize the intricacy of designing a reliable placebo control group. Nonetheless, our study revealed that different sham groups, along with their underlying placebo effects, could potentially interfere with the interpretation of manual therapy studies. We identified significant differences in the designs employed for placebo groups, contributing to the high heterogeneity of the results. Additionally, we have brought to light the absence of participant expectation assessment in the majority of the trials, which could be pivotal in establishing reliable placebo control groups. We also note the significance of incorporating the use of TIDier-Placebo [5], as suggested in the comment by Giacomo Rossettini et al., to further enhance the quality and transparency of future studies.

In relation to the specific syntax implemented for each database, although an explicit request for the exact search strategy was not made, we have provided it in this comment (Table 1). Furthermore, we are more than willing to share all the data used in our study with the authors or any other interested individuals.

## Figures and Tables

**Table 1:** Search strategies for all included search engines.

EMBASE (ELSEVIER)	
1.	‘musculoskeletal manipulation’ OR ‘manipulative medicine’ OR (neural AND tension AND technique) OR ‘neural mobilization’ OR ‘neural mobilisation’ OR ‘neural stretching’ OR ‘neural gliding’ OR ‘massage’ OR ‘soft tissue techniques’ OR ‘ischemic compression’
2.	‘placebo’ OR ‘sham procedure’
3.	‘pain’
4.	#1 AND #2 AND #3
CINAHL (EBSCO)	
1.	“musculoskeletal manipulations” OR “manual therap*” OR “manipulation*” OR “mobilization*” OR “mobilisation*” OR “neural tension technique*” OR “neural mobilization*” OR “neural mobilisation” OR “neural stretching” OR “neural gliding” OR “massage*” OR “massotherapy” OR “soft tissue techniques” OR “inhibition technique*” OR “ischemic compression”
2.	placebo* OR sham
3.	pain
4.	#1 AND #2 AND #3
PsycINFO (EBSCO)	
1.	“musculoskeletal manipulations” OR “manual therap*” OR “manipulation*” OR “mobilization*” OR “mobilisation*” OR “neural tension technique*” OR “neural mobilization*” OR “neural mobilisation” OR “neural stretching” OR “neural gliding” OR “massage*” OR “massotherapy” OR “soft tissue techniques” OR “inhibition technique*” OR “ischemic compression”
2.	placebo* OR sham
3.	pain
4.	#1 AND #2 AND #3
Medline (EBSCO)	
1.	“musculoskeletal manipulations” OR “manual therap*” OR “manipulation*” OR “mobilization*” OR “mobilisation*” OR “neural tension technique*” OR “neural mobilization*” OR “neural mobilisation” OR “neural stretching” OR “neural gliding” OR “massage*” OR “massotherapy” OR “soft tissue techniques” OR “inhibition technique*” OR “ischemic compression”
2.	placebo* OR sham
3.	pain
4.	#1 AND #2 AND #3
PubMed (NLM)	
1.	“musculoskeletal manipulations” [Mesh] OR “manual therap*” OR “manipulation*” OR “mobilization*” OR “mobilisation*” OR “neural tension technique*” OR “neural mobilization*” OR “neural mobilisation” OR “neural stretching” OR “neural gliding” OR “massage*” OR “massotherapy” OR “soft tissue techniques” OR “inhibition technique*” OR “ischemic compression”)
2.	placebo* OR sham
3.	pain
4.	#1 AND #2 AND #3
Scopus (ELSEVIER)	
1.	TITLE-ABS-KEY (“musculoskeletal manipulations” OR “manual therap*” OR “manipulation*” OR “mobilization*” OR “mobilisation*” OR “neural tension technique*” OR “neural mobilization*” OR “neural mobilization” OR “neural stretching” OR “neural gliding” OR “massage*” OR “massotherapy” OR “soft tissue techniques” OR “inhibition technique*” OR “ischemic compression”)
2.	TITLE-ABS-KEY (sham OR placebo*)
3.	TITLE-ABS-KEY (pain)
4.	#1 AND #2 AND #3
Web of Science (ELSEVIER)	
1.	TS= “musculoskeletal manipulations” OR “manual therap*” OR “manipulation*” OR “mobilization*” OR “mobilisation*” OR “neural tension technique*” OR “neural mobilization*” OR “neural mobilisation” OR “neural stretching” OR “neural gliding” OR “massage*” OR “soft tissue techniques” OR “inhibition technique*” OR “ischemic compression”
2.	TS= “sham” OR “placebo”
3.	TS= “pain”
4.	#1 AND #2 AND #3
PEDro	
1.	Abstract & Title: shamTherapy: stretching, mobilization, manipulation, massageProblem: painMethod: Clinical TrialWhen searching: AND
2.	Abstract & Title: placeboTherapy: stretching, mobilization, manipulation, massageProblem: painMethod: Clinical TrialWhen searching: AND
The Cochrane Library	
1.	MeSH descriptor: [Musculoskeletal Manipulations] explode all trees OR (manual therap*):ti,ab,kw OR (manipulation*):ti,ab,kw OR (mobilization*):ti,ab,kw OR (mobilisation*):ti,ab,kw OR (neural tension technique*):ti,ab,kw OR (neural mobilization*):ti,ab,kw OR (neural mobilisation*):ti,ab,kw OR (neural stretching):ti,ab,kw OR (neural gliding):ti,ab,kw OR (massage):ti,ab,kw OR (massotherapy):ti,ab,kw OR (soft tissue techniques):ti,ab,kw OR (inhibition technique*):ti,ab,kw OR (ischemic compression):ti,ab,kw
2.	(sham):ti,ab,kw OR (placebo*):ti,ab,kw
3.	(pain):ti,ab,kw
4.	#1 AND #2 AND #3

Truncation with the (*) in databases was used for the broadening of search queries, encompassing various words.
